# Lymphatic Drainage Patterns from Melanomas on the Shoulder or Upper Trunk to Cervical Lymph Nodes and Implications for the Extent of Neck Dissection

**DOI:** 10.1245/s10434-012-2387-2

**Published:** 2012-05-11

**Authors:** Hidde J. Veenstra, W. Martin C. Klop, Maarten J. Speijers, Peter J. F. M. Lohuis, Omgo E. Nieweg, Harald J. Hoekstra, Alfons J. M. Balm

**Affiliations:** 1 Department of Head and Neck Surgery and Oncology, The Netherlands Cancer Institute – Antoni van Leeuwenhoek Hospital, Amsterdam, The Netherlands; 2 Department of Otolaryngology, Academic Medical Centre, Amsterdam, The Netherlands; 3 Department of Surgery, The Netherlands Cancer Institute—Antoni van Leeuwenhoek Hospital, Amsterdam, The Netherlands; 4 Department of Surgery, University Medical Centre Groningen, University of Groningen, Groningen, The Netherlands

## Abstract

**Purpose:**

To determine the incidence and pattern of cervical lymphatic drainage in patients with melanomas located on the upper limb or trunk, and to evaluate our current neck dissection protocol for those patients with a N+ neck.

**Methods:**

Of 1192 melanoma patients who underwent sentinel node biopsy, 631 were selected with a primary tumor on the upper limb or trunk. All lymphoscintigrams, SPECT/CT images and operative reports were reviewed to determine the exact locations of sentinel nodes visualized preoperatively and dissected during operation.

**Results:**

Thirty-nine (6.2 %) of 631 patients with a melanoma on the upper limb or trunk showing cervical lymph node drainage were identified. In 34 (87 %) of 39 patients, sentinel nodes were excised from level IV or Vb, and in 30 of those 39 patients simultaneous from the axilla. In the remaining five patients (13 %), sentinel nodes were collected from level IIb, level III or the suboccipital region. All collected sentinel nodes were located in the intended dissection area for N+ patients. Thirteen patients (33 %) had a total of 22 tumor-positive sentinel nodes in either the axilla (*n* = 10), level IV (*n* = 2), Vb (*n* = 9) or suboccipital (*n* = 1).

**Conclusions:**

Only a minority of the patients with upper limb or trunk melanomas demonstrated lymphatic drainage to cervical lymph node basins, with preferential drainage to levels IV and Vb. Our current dissection protocol of levels II–V, with or without extension to the suboccipital region, in those patients with involved cervical sentinel nodes seems sufficient.

Sentinel node biopsy is increasingly used to stage patients with melanoma and has evolved into a routine procedure for tumors originating at different sites of the body.^1^ The head and neck region is known for its complex lymphatic drainage, with melanomas draining to multiple, bilateral and sometimes even contralateral sentinel nodes.^2^–^5^ The high number of lymph nodes and lymphatic vessels in a small anatomic area may contribute to this phenomenon.^6^ However, despite the complex drainage pathways, head and neck melanomas usually seem to follow a somewhat predictable pattern, with preferred drainage to the parotid and levels I–V for lesions located anterior of the so-called watershed line and to the suboccipital region and level II–V for tumors located posterior of this line.^7^–^9^ On the basis of the primary tumor location, O’Brien et al.^10^ defined the extent of several therapeutic neck dissections.

Occasionally, primary melanomas located on the upper limb or trunk drain to cervical lymph nodes, but studies on that subject are scarce.^11^–^13^ Uren et al. presented 1735 patients with melanomas located on the upper limb or upper trunk where lymphatic drainage was visualized to a total number of 321 cervical sentinel nodes.^13^ A completion neck dissection in patients with a histologically proven tumor-positive sentinel node originating from a melanoma on the upper limb or trunk is usually advised. The extent of therapeutic neck dissection hereby is based on the expected drainage pathways and generally includes levels II–V (with or without the suboccipital region). However, in these specific cases the actual extent of the neck dissection is still a matter of ongoing debate, because scientific evidence for this approach is lacking. Therefore, the aim of this study is to determine the incidence of lymphatic drainage to cervical lymph nodes derived from melanomas located on the upper limb or trunk in our patient group, and to evaluate our current neck dissection protocol of the levels II–V (with or without the suboccipital region) in these patients with a N+ neck.

## Patients and Methods

### Patients

From 1192 unselected melanoma patients who underwent sentinel node biopsy at the Netherlands Cancer Institute or the University Medical Centre Groningen between December 1991 and July 2010 a total of 631 patients with a primary melanoma on the upper limb or trunk were identified. The anterior trunk melanomas were located on the trunk from the umbilicus up to the clavicle and on the anterior side of the shoulder, which is divided by an imaginary line over the deltoid region. The posterior trunk melanomas were located on the trunk from the level of the umbilicus up to vertebra C7 and on the posterior side of the shoulder posterior from the imaginary line. Sentinel node biopsy was carried out if the primary tumor had a Breslow thickness of at least 1 mm or a Clark level IV.

### Imaging

On the day before operation, technetium-99m-labeled nanocolloid (Nanocoll, Amersham Cygne, Eindhoven, The Netherlands) was injected intradermally around the biopsy site in a mean volume of 0.4 ml and a mean dosage of 63 MBq (2 mCi). The mean time interval between injection of the technetium-99m-labeled nanocolloid and the actual operation is approximately 18 hours. Static images were performed and were preceded by a dynamic study of ten minutes. Both anterior and lateral images were routinely made. Immediate and three-hour postinjection lymphoscintigrams were evaluated with regard to the location, number and sequence of appearance of sentinel nodes. At The Netherlands Cancer Institute, hybrid single-photon emission computed tomography with CT (SPECT/CT) was introduced in 2006 and was performed when conventional images were difficult to interpret and for research purposes.^14^ SPECT/CT was additionally performed in eight patients, all treated in the Netherlands Cancer Institute. A sentinel node was defined as a lymph node upon which the primary tumor drains directly.^15^ Both conventional lymphoscintigrams and SPECT/CT images were used to mark the locations of the sentinel nodes on the skin with indelible ink.

### Surgical and Pathological Procedures

Lymphoscintigrams and SPECT/CT images were discussed with the surgeon before the sentinel nodes were excised. The next day, patent blue dye (Laboratoire Guerbet, Aulnay-Sous-Bois, France) was administered intradermally in a mean volume of 1.0 ml, completely surrounding the scar of the reexcision site. Ten minutes after injection all nodal basins identified by lymphoscintigraphy were explored surgically through limited incisions. Surgical dissection was guided by a handheld gamma ray detection probe (Neoprobe 1000 and 1500, Johnson & Johnson Medical, Hamburg, Germany) and by looking for blue-stained afferent lymphatic vessels that led to blue stained sentinel lymph nodes. Once the sentinel node had been excised, the probe was used to search the resection bed to ensure that there were no residual sentinel nodes. The search for additional sentinel nodes was ceased if there was no residual activity or if this was less than ten percent of the most active node in the same area. After sentinel node biopsy, wide local excision was performed of the primary melanoma site with a 1 or 2 cm margin, depending on the Breslow thickness. All sentinel nodes were formalin-fixed, bisected, paraffin-embedded, and cut at a minimum of six levels at 50 to 150 μm intervals. Pathologic evaluation included hematoxylin and eosin and immunohistochemical staining (S-100 and MART-1). With the exception of one patient harboring a small tumor burden in the sentinel node (Starz classification I), patients with a tumor-positive sentinel node underwent therapeutic node dissection.^16^–^18^ Patients received a level I–III axillary dissection for tumor-positive sentinel nodes located in the axilla. In case a cervical positive sentinel node was collected, derived from an anterior shoulder or anterior trunk melanoma, a selective neck dissection of the levels II–V was performed. The dissection was extended to the suboccipital region in case a tumor-positive sentinel node was found in level II–V or the suboccipital region derived from a melanoma on the posterior shoulder or posterior trunk. Extension to the suboccipital region consists of the ‘so-called’ hockey-stick incision more posteriorly for a posterolateral neck dissection (levels II–V including the suboccipital region). During suboccipital dissection a thin layer of fat and lymph nodes between the ligamentum nuchae (medial border) and the fascia of trapezius muscle (deep border) is removed. The supreme nuchal line and external occipital protuberance represent the cranial border of the dissection and an imaginary line connecting the two mastoid processes the inferior border. The suboccipital dissection is performed in continuity with the selective neck dissection (II–V).^19^,^20^ Dissection of both the axilla and neck was performed if metastases were present at each site in the same patient. All lymph nodes collected from regional node dissection specimens were examined in 4.0 mm sections stained with hematoxylin and eosin.

### Data Analysis

To determine the precise location of sentinel nodes visualized preoperatively and collected during operation all lymphoscintigrams, SPECT/CT images and operative reports were retrospectively reviewed. All patients were monitored with a median follow-up of 54 months.

## Results

### Patients

Thirty-nine (6.2 %) of 631 patients with a primary melanoma located either on the upper limb or trunk were identified with lymphatic drainage to at least one lymph node basin in the head and neck area. All melanomas on the upper limb were located on the anterior or posterior shoulder. Overall patient characteristics are shown in Table 1.

**Table 1:** Characteristics of 39 patients with a melanoma on the upper limb or trunk

Characteristic	Value
Age (y), mean (range)	56 (15–89)
Gender (M:F)	27:12
Primary tumor location	
Anterior shoulder	3
Posterior shoulder	12
Anterior trunk	5
Posterior trunk	19
Breslow thickness (mm), median (range)	2.9 (1.0–12)
Follow-up (mo), median (range)	54 (4–162)

### Lymphoscintigraphy and SPECT/CT

Both conventional lymphoscintigraphy and SPECT/CT visualized a total of 112 sentinel nodes in 39 patients. Forty-four sentinel nodes were depicted in the axilla, 67 sentinel nodes in cervical lymph node basins and one sentinel node was seen on the lateral border of the left scapula. In 31 (80 %) of 39 patients lymphatic drainage from the shoulder or upper trunk was seen to level Vb, of which 27 patients had simultaneous drainage to the axilla. Two patients (5 %) showed lymphatic drainage to level IV of which one also to the axilla. In the remaining six patients (15 %), lymphatic drainage was depicted to the parotid gland, level Va, level III, level IIa, level IIb or suboccipital.

### Sentinel Node Biopsy

**Fig. 1:**
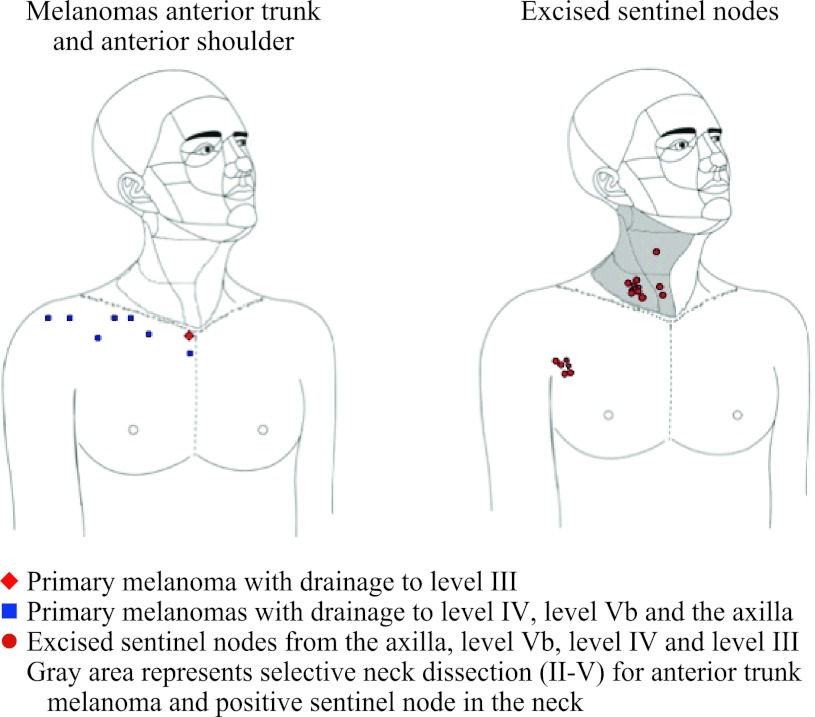
Locations of eight primary melanomas on the anterior shoulder and anterior trunk and 24 sentinel nodes draining these primary lesions. In *gray* the dissection area is visualized in case a tumor-positive sentinel node is collected

**Fig. 2:**
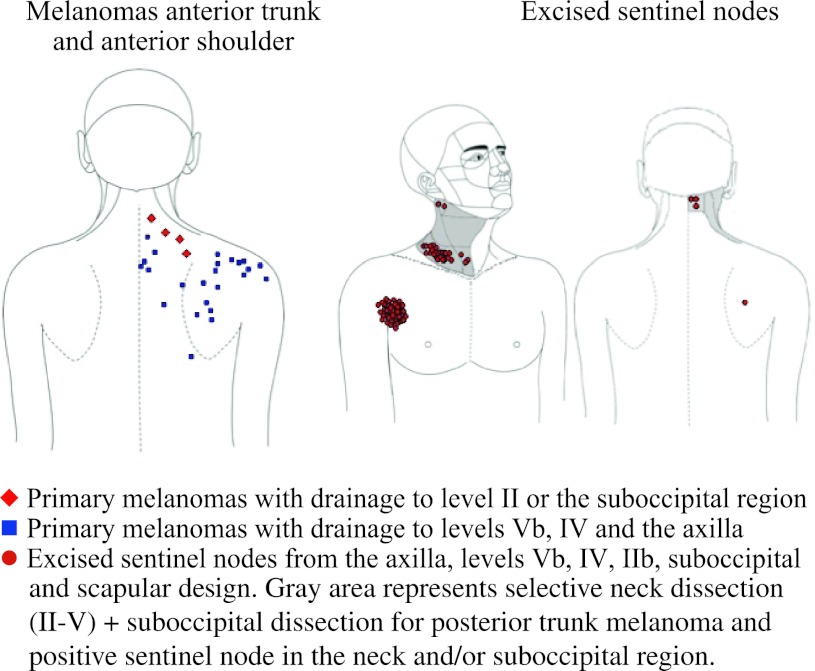
Locations of 31 primary melanomas on the posterior shoulder and posterior trunk and 97 sentinel nodes draining these primary lesions. In *gray* the dissection area is visualized in case a tumor-positive sentinel node is collected

In 39 patients a total of 121 sentinel nodes were excised (Table 2). Forty-four sentinel nodes were collected from the axilla, 76 sentinel nodes from cervical lymph node basins and one sentinel node from the lateral border of the left scapula (Figs. 1, 2). In 32 (82 %) of 39 patients, a total of 65 sentinel nodes were excised from level Vb. In 28 of those 39 patients, 44 sentinel nodes were also excised from the axilla or lateral border of the left scapula. In two patients (5 %), five sentinel nodes were collected from level IV, of which one also had a sentinel node in the axilla. In the remaining five patients (13 %), two sentinel nodes were excised from level IIb, one sentinel node from level III and three sentinel nodes from the suboccipital region. In two patients, these suboccipital nodes were not visualized preoperatively, but the handheld gamma probe detected them. All collected suboccipital sentinel nodes were found in patients with a primary tumor located on the upper posterior trunk or posterior shoulder. Not all preoperatively visualized sentinel nodes were collected. A sentinel node in the parotid gland, in level Va and in level IIa was not found during operation, because the handheld gamma probe did not detect any radioactivity.

**Table 2:** Locations of sentinel nodes excised

Melanoma site	No. of patients	Location								
		Axilla	Level IIa	Level IIb	Level III	Level IV	Level Va	Level Vb	Suboccipital	Other
Anterior shoulder	3	4 (1)	–	–	–	–	–	5 (1)	–	
Anterior trunk	5	3 (1)	–	–	1 (0)	2 (0)	–	9 (0)	–	
Posterior shoulder	12	15 (2)	–	1 (0)	–	–	–	20 (5)	1 (1)	
Posterior trunk	19	22 (6)	–	1 (0)	–	3 (2)	–	31 (3)	2 (0)	Scapula
Total	39	44 (10)	–	2 (0)	1 (0)	5 (2)	–	65 (9)	3 (1)	1 (0)

The total number of tumor-positive sentinel nodes found is indicated in parentheses

### Pathology

In 13 (33 %) of the 39 patients, a total of 22 tumor-positive sentinel nodes were excised. The involved sentinel nodes were located in the axilla, level IV, level Vb or suboccipital (Table 3). One patient did not receive any further treatment, because only a minor tumor burden (Starz I) was found in the involved sentinel node.^16^–^18^ In two (17 %) of 12 patients who underwent a regional lymph node dissection, additional tumor-positive lymph nodes were collected. The first patient with a melanoma on the posterior shoulder underwent a posterolateral dissection with extension to the suboccipital region and one additional tumor-positive lymph node was found in level Vb. The second patient with a melanoma on the scapula underwent both an axillary dissection and a posterolateral dissection with extension to the suboccipital region. Two additional tumor-positive lymph nodes were excised from the axilla and two additional tumor-positive lymph nodes from level Vb (Table 3). Both patients received adjuvant radiotherapy.

**Table 3:** Data of 12 patients who underwent therapeutic lymph node dissection

Patient no.	Tumor location	Location of positive sentinel nodes	Type of therapeutic node dissection	Positive lymph nodes in dissection specimen
1	Posterior trunk	Axilla	Axillary dissection	0/15
2	Posterior shoulder	Axilla; level Vb	Axillary dissection; posterolateral dissection level II–V including the suboccipital region	0/17; 0/13
3	Anterior shoulder	Level Vb	Axillary dissection; selective dissection level II–V	0/35
4	Posterior trunk	Axilla; suboccipital	Axillary dissection; posterolateral dissection level II–V including the suboccipital region	0/17; 0/19
5	Posterior trunk	Axilla	Axillary dissection	0/11
6	Posterior trunk	Axilla; level Vb	Axillary dissection; posterolateral dissection level II–V including the suboccipital region	2/16; 2/53 (level Vb)
7	Posterior shoulder	Axilla; level Vb	Axillary dissection; selective dissection level II–V	0/42; 0/84
8	Posterior shoulder	Level Vb	Posterolateral dissection level II–V including the suboccipital region	0/13
9	Posterior shoulder	Axilla; level Vb	Axillary dissection; posterolateral dissection level II–V including the suboccipital region	0/14; 0/21
10	Posterior shoulder	Level Vb	Posterolateral dissection level II–V including the suboccipital region	1/37 (level Vb)
11	Posterior trunk	Level IV	Posterolateral dissection level II–V including the suboccipital region	0/32
12	Posterior trunk	Axilla; level Vb	Axillary dissection; posterolateral dissection level II–V including the suboccipital region	0/14; 0/16

### Follow-up

Three of the 26 patients with a tumor-negative sentinel node developed a recurrence, with an average follow-up of 44 (range 19–162) months. The first patient, who had a primary tumor on the left shoulder, developed a subcutaneous metastasis on the left wrist and simultaneously pulmonary metastases after 107 months and is currently being treated with systemic therapy. The second patient with a primary melanoma on the upper posterior trunk developed pulmonary metastases after 32 months and died due to these metastases 3 months later. The third patient with a primary tumor on the right shoulder and tumor-negative sentinel nodes removed from level IIb and Vb developed a lymph node recurrence after 5 months in the previously biopsied level Vb and this procedure was thus considered falsely negative. After therapeutic neck dissection no other recurrences developed in the next 5 years. Eight of 12 tumor-positive sentinel node patients who underwent therapeutic dissection remained disease free, with an average follow-up of 59 months (range, 17–150). Four of 12 patients with a tumor-positive sentinel node, who received therapeutic lymph node dissection, developed a local recurrence. In none of these latter four patients additional tumor-positive lymph nodes were found in the dissection specimen. The first patient developed extensive distant metastases after 31 months and died from these recurrences. The second patient developed brain and oropharynx metastases after 24 months and is still alive 4 months later. In the third patient liver metastases were found 9 months after neck dissection and this patient died 3 months later due to these metastases. The fourth developed patient a local recurrence in the scar of the primary tumor excision 48 months after sentinel node biopsy, which was reexcised and no further recurrences developed. The patient with a tumor-positive sentinel node who received no further treatment (Starz I) remained disease free with a follow-up of 25 months. No lymph node recurrences developed beyond the dissected neck levels in patients who underwent a neck dissection.

## Discussion

This study demonstrates 39 patients with a melanoma on the shoulder or upper trunk with drainage to at least one cervical lymph node basin. Thirty-four (87 %) of these 39 patients had lymphatic drainage to levels IV and Vb, and the axilla. In the remaining five patients (13 %), sentinel nodes were collected from level IIb, level III or from the suboccipital region. As our current dissection protocol for patients with a tumor-positive sentinel node consist of level II–V for melanomas located on the anterior shoulder and anterior trunk and of level II–V with extension to the suboccipital region for melanomas on the posterior shoulder and posterior trunk, 100 % of the collected cervical sentinel nodes are covered.

The lymphatic drainage pattern from primary melanomas located on the shoulder or trunk to cervical lymph node basins can’t be compared with the lymphatic drainage pattern of head and neck melanomas. So far, no studies have been published dealing with this specific subject. Reynolds et al. mapped lymphatic drainage of 5239 melanoma patients.^12^,^21^ In total, 2406 patients had melanomas located on the trunk (above the waist or umbilicus) or upper limb. Although the incidence of patients with cervical lymph drainage was not reported, it could be calculated that a total of 569 sentinel nodes were visualized in cervical lymph node basins. A total of 508 (89 %) of 569 sentinel nodes were visualized in level Vb (*n* = 330) and level Va (*n* = 178), 30 sentinel nodes (5.5 %) in level IV, 15 sentinel nodes (2.5 %) in level III and 11 sentinel nodes (2 %) in level II. In contrast to our findings, two sentinel nodes (0.5 %) were visualized in level I derived from melanomas located just infraclavicularly. Three sentinel nodes (0.5 %) were found in the suboccipital region, originating from melanomas located on the upper posterior trunk and posterior shoulder. In the present study 76 cervical sentinel nodes were identified in the 39 included patients. It has to be emphasized that the results of Reynold’s study were based on preoperative lymphoscintigrams without confirmation by additional dissection, as we did. The definite locations of the sentinel node might therefore be different, as has been shown by Jansen et al.^5^ In their study, a discrepancy between sentinel node locations depicted by preoperative imaging and collected during operation was shown in 18 (60 %) of 30 patients. The current study demonstrated this inconsistency in 6 (15 %) of 39 patients. For example, in one patient a sentinel node was depicted in the parotid preoperatively, but was collected from level IIb.

Melanoma cells detected in cervical sentinel nodes derived from tumors on the shoulder or trunk are considered as regional metastases and therefore, outside a clinical trial, completion neck dissection is advised. The extent of neck dissection, as described in the methods section, is based on the principle that only those lymph node basins considered at risk for metastases should be removed. A total of twelve patients with a primary melanoma located on the shoulder or trunk underwent completion lymph node dissection (Table 3) and in two patients (17 %) additional tumor-positive lymph nodes were found in level Vb and the axilla. The percentage of additional melanoma containing lymph nodes in the head and neck region is comparable with the percentages reported by other authors of 10–30 %.^8^,^22^–^24^ The safety of our current neck dissection protocol for patients with tumor-positive cervical sentinel nodes derived from shoulder or trunk melanoma is also confirmed by the lack of regional recurrences developing outside the operation field. This even could raise the question if a more selective approach guided by the lymph node drainage pattern is appropriate, for example, by including only the first- and second echelon nodes in the dissection area. Comparable to Reynolds et al., we demonstrated that the vast majority of sentinel nodes are located in level IV and Vb. Although only one tumor-positive sentinel node was found outside these levels in the suboccipital region, lymphatic drainage was also shown to level II and III. On the basis of these results, and the knowledge that anatomic boundaries of the cranial border of level IV and Vb are hard to define, it seems adequate to continue our current neck dissection protocol including level II–V (with or without extension to the suboccipital region) in the N+ patients with primary melanomas located on the shoulder or trunk.^25^

As in our study, Reynolds et al.^12^ showed lymphatic drainage to the suboccipital region in three patients with a melanoma located on the upper posterior trunk or posterior shoulder. In the current study one patient had a tumor-positive lymph node in the suboccipital area and underwent a level II–V dissection with extension to the suboccipital region. There were no involved nonsentinel nodes in the suboccipital dissection specimens, but drainage to the suboccipital region was seen in three of the 31 patients (10 %) with a posteriorly located melanoma. This demonstrates that the suboccipital region remains a potential drainage site for metastatic cells. In our opinion, this observation justifies treatment of the suboccipital lymph node basin for melanomas of the posterior shoulder and upper back in the N+ neck. Nevertheless the absence of a clear surgical boundary of this region and relatively increased risk of flap necrosis (a thin skin flap has to be elevated because the suboccipital nodes are mainly located just under the skin) makes it a more delicate procedure. The suboccipital dissection can be omitted if the risk of morbidity is considered to exceed the potential benefit. Strict follow-up of this specific area by ultrasound is then recommended.

In conclusion, in our study, 39 patients are described with a primary melanoma located on the shoulder or upper trunk with lymphatic drainage to cervical lymph node basins. The preferred drainage pattern was to level IV, level Vb and the axilla, but in some cases lymphatic flow was seen up to level II, level III or the suboccipital region. Our current dissection protocol of the levels II–V, in case a tumor-positive cervical sentinel node is collected in patients with a melanoma on the anterior shoulder or anterior upper trunk, appears to be sufficient. For patients with an involved cervical sentinel node originating from a primary melanoma on the posterior upper trunk or posterior shoulder, a level II–V dissection with extension to the suboccipital region is advised.

## References

[1] Balch CM, Morton DL, Gershenwald JE (2009). Sentinel node biopsy and standard of care for melanoma. J Am Acad Dermatol..

[2] De Wilt JH, Thompson JF, Uren RF (2004). Correlation between preoperative lymphoscintigraphy and metastatic nodal disease sites in 362 patients with cutaneous melanomas of the head and neck. Ann Surg..

[3] O’Brien CJ, Uren RF, Thompson JF (1995). Prediction of potential metastatic sites in cutaneous head and neck melanoma using lymphoscintigraphy. Am J Surg..

[4] Chao C, Wong SL, Edwards MJ (2003). Sentinel lymph node biopsy for head and neck melanomas. Ann Surg Oncol..

[5] Jansen L, Koops HS, Nieweg OE (2000). Sentinel node biopsy for melanoma in the head and neck region. Head Neck..

[6] Pan WR, Suami H, Taylor GI (2008). Lymphatic drainage of the superficial tissues of the head and neck: anatomical study and clinical implications. Plast Reconstr Surg..

[7] Klop WM, Veenstra HJ, Vermeeren L (2011). Assessment of lymphatic drainage patterns and implications for the extent of neck dissection in head and neck melanoma patients. J Surg Oncol..

[8] Lin D, Franc BL, Kashani-Sabet M (2006). Lymphatic drainage patterns of head and neck cutaneous melanoma observed on lymphoscintigraphy and sentinel lymph node biopsy. Head Neck..

[9] Pathak I, O’Brien CJ, Petersen-Schaeffer K (2001). Do nodal metastases from cutaneous melanoma of the head and neck follow a clinically predictable pattern?. Head Neck..

[10] O’Brien CJ, Petersen-Schaefer K, Ruark D (1995). Radical, modified, and selective neck dissection for cutaneous malignant melanoma. Head Neck..

[11] Wagner JD, Park HM, Coleman JJ (2000). Cervical sentinel lymph node biopsy for melanomas of the head and neck and upper thorax. Arch Otolaryngol Head Neck Surg..

[12] Reynolds HM, Dunbar PR, Uren RF (2007). Three-dimensional visualisation of lymphatic drainage patterns in patients with cutaneous melanoma. Lancet Oncol..

[13] Uren RF, Howman-Giles R, Thompson JF (2003). Patterns of lymphatic drainage from the skin in patients with melanoma. J Nucl Med..

[14] Van der Ploeg IM, Valdés Olmos RA, Kroon BBR (2009). The yield of SPECT/CT for anatomical lymphatic mapping in patients with melanoma. Ann Surg Oncol..

[15] Nieweg OE, Tanis PJ, Kroon BBR (2001). The definition of a sentinel node. Ann Surg Oncol..

[16] Van der Ploeg IM, Kroon BBR, Antonini N (2009). Is completion lymph node dissection needed in case of minimal melanoma metastasis in the sentinel node?. Ann Surg..

[17] Van der Ploeg IM, Kroon BBR, Antonini N (2009). Comparison of three micromorphometric pathology classifications of melanoma metastases in the sentinel node. Ann Surg..

[18] Starz H, Siedlecki K, Balda BR (2004). Sentinel lymphonodectomy and s-classification: a successful strategy for better prediction and improvement of outcome of melanoma. Ann Surg Oncol..

[19] Diaz EM, Austin JR, Burke LI, Goepfert H (1996). The posterolateral neck dissection. Technique and results. Arch Otolaryngol Head Neck Surg..

[20] Thiel W (1999). Photographic atlas of practical anatomy II.

[21] Reynolds HM, Smith NP, Uren RF (2009). Three-dimensional visualization of skin lymphatic drainage patterns of the head and neck. Head Neck..

[22] De Rosa N, Lyman GH, Silbermins D (2011). Sentinel node biopsy for head and neck melanoma: a systematic review. Otolaryngol Head Neck Surg..

[23] Gomez-Rivera F, Santillan A, McMurphey AB (2008). Sentinel node biopsy in patients with cutaneous melanoma of the head and neck: recurrence and survival study. Head Neck..

[24] Carlson GW, Murray DR, Lyles RH (2005). Sentinel lymph node biopsy in the management of cutaneous head and neck melanoma. Plast Reconstr Surg..

[25] Lohuis PJ, Klop WM, Tan IB (2004). Effectiveness of therapeutic (N1, N2) selective neck dissection (levels II to V) in patients with laryngeal and hypopharyngeal squamous cell carcinoma. Am J Surg..

